# Vulnerability to Psychosis: A Psychoanalytical Perspective. The Paradigmatic Example of 22q11.2 Microdeletion Syndrome

**DOI:** 10.3389/fpsyg.2020.01613

**Published:** 2020-09-15

**Authors:** Rémy Potier, Sarah Troubé, Olivier Putois

**Affiliations:** ^1^Université Paris Diderot, Paris, France; ^2^Département des Études Psychanalytiques, Institut des Sciences, Humaines, des Sciences et des Sociétés, Université Paris Diderot, Paris, France; ^3^Laboratoire Interdisciplinaire Récits Cultures Et Sociétés (LIRCES EA 3159), Université Nice-Sophia Antipolis, Nice, France; ^4^Subjectivité, Lien Social et Modernité, Faculté de Psychologie, Université de Strasbourg, Strasbourg, France; ^5^Service de Psychiatrie, Santé Mentale et Addictologie, CHU de Strasbourg, Strasbourg, France

**Keywords:** 22q11.2 microdeletion syndrome, neurodevelopmental genetic disorders, psychotic vulnerability, schizophrenia, anxiety disorders, developmental trajectory, psychodynamics of anxiety, psychoanalytic approach to differential diagnosis

## Abstract

This paper outlines a psychoanalytic contribution to a growing research field in psychiatry: that of psychotic vulnerability, and the related neurogenetic modeling of schizophrenia. We explore this contribution by focusing on recent studies concerning a neurodevelopmental disorder, the 22q11.2 microdeletion syndrome – which comprises DiGeorge syndrome in particular. It is one of the most common rare genetic syndromes, and the patients that it affects present a very high rate of psychotic symptoms (between 30 and 40%). For this reason, it has sparked an increasing number of clinical research projects which give it a paradigmatic status, as much for psychotic vulnerability as for potential neurobiological and genetic markers of schizophrenia. This syndrome illustrates one of the major stakes in contemporary psychopathology: the articulation of clinical, neurocognitive, and genetic approaches in a pluri-disciplinary manner. We seek to show that psychoanalysis, when it participates in this articulation, opens up specific hypotheses and research perspectives. In particular, based on the epidemiological observation of the role of anxiety as a predictor for psychosis, we underline the potential relevance of psychoanalytically oriented differential clinical practice and the psychodynamics of anxiety: they can contribute to studies and clinical follow-up on the 22q11.2 microdeletion syndrome and, more widely, to research on the detection and prevention of psychotic vulnerability.

## Introduction

Contemporary psychopathology presents a growing number of psychiatric studies focused on risk factors for psychosis and its prevention (for a historical perspective and contemporary overview, see Lieberman et al., 2019). Innumerable epidemiological studies have sought to identify clinical predictors along with genetic, neurobiological and cognitive correlates associated with schizophrenia (Yung et al., 2004; Fusar-Poli et al., 2013; Sass, 2014). These papers have major implications for the definition and etiological models of psychotic disorders, and for the clinical follow-up of subjects at risk of schizophrenia.

We believe that a psychoanalytic approach could contribute to this field by drawing on recent studies focusing on a neuro-developmental pathology: 22q11.2 microdeletion syndrome. Identified in the 1990s as DiGeorge syndrome then velocardiofacial syndrome (VCFS), the genetic origin of which lies in a microdeletion located on chromosome 22q11.2 (Scambler et al., 1991), this syndrome is often under-diagnosed: according to recent studies (McDonald-McGinn et al., 2015), it affects one birth in 1000–4000. Schizophrenia seemingly arises in 30% of adolescents and young adults, and psychotic episodes are prevalent in as many as 40%. This syndrome is therefore viewed as paradigmatic for vulnerability to psychosis, and as allowing the development of an integrative model for psychotic vulnerability, at the intersection between child psychiatry and neuro-genetic psychiatry.

The importance of anxiety as a risk factor for psychosis in these patients opens the possibility of a psychoanalytical contribution. Viewed from this perspective, recent studies on the syndrome lead to question the different clinical forms of anxiety and their role in differential diagnosis: questions that are central in psychoanalytical typologies and hypotheses. Thus, a psychoanalytic perspective could provide material to address the lack of sensitive and specific diagnostic criteria for pre-syndromal schizophrenia faced by contemporary approaches (cf. Lieberman et al., 2019).

Thus, we could combine the contributions of two research fields to study the 22q11.2 microdeletion, for which the pertinence of a psychodynamic point of view has already been sketched out: that of clinical practice with neurodevelopmental disorders (Ouss et al., 2014) and that of vulnerability to psychosis (Evrard, 2011; Troubé, 2013; De Masi, 2018).

## Anxiety, a Risk Factor for Psychosis in 22q11.2 Microdeletion

The 22q11.2 microdeletion syndrome generates highly polymorphous (Swillen and McDonald-McGinn, 2015) clinical manifestations, bringing together somatic, neurocognitive and psychopathological disturbances. Organic anomalies in particular affect the heart, the endocrine, and immune systems, as well as the face, leading to disturbances of deglutition and vocal articulation. Psychomotor difficulties are frequently associated with delayed speech development and moderate intellectual deficit, as well as attention deficit and social cognition deficit (Philip and Bassett, 2011; Angkustsiri et al., 2014). The comorbidity with psychopathological disorders is considerable: 22q11.2 children display a high frequency of attention deficit hyperactivity disorder (35–40%), anxiety disorders (50–55%), and social withdrawal and oppositional defiant disorder (35–40%) (Jolin et al., 2009). In adulthood, the prevalent mental disorders are psychotic disorders (30%), anxiety disorders (25–30%), and mood disorders (15–20%) (Angkustsiri et al., 2014; Schneider et al., 2014, 2016, 2019). Furthermore, it is not rare for psychotic symptoms to appear before adolescence (Debbané et al., 2006; Tang et al., 2014, 2017; Tang and Gur, 2018) and for psychotic prodromes to be correlated with schizotypal traits (Fonseca-Pedrero et al., 2016).

These recent longitudinal studies stress a correlation between the first symptomatic signs of psychosis and the presence of anxiety disorders during earlier developmental trajectory (Antshel et al., 2010; Gothelf et al., 2013; Schonherz and Davidov, 2014; Stephenson et al., 2015). The strong prevalence of psychotic disorders in this syndrome could also be linked to the frequency of anxiety disorders in childhood. 22q11.2 microdeletion syndrome would then confirm previous results underlining a higher risk of psychosis among subjects with anxiety disorders in the general population (Tien and Eaton, 1992). In this sense, the syndrome would reveal more general mechanisms potentially at play in the onset of psychotic disorders.

However, clinical practice with patients afflicted with this syndrome also seems to shed light on questions raised by the very notion of psychotic vulnerability, which could benefit from a psychoanalytical clarification.

First of all, if anxiety disorders indicate vulnerability, to what extent would this vulnerability be specific to schizophrenia? Clinically speaking, anxiety disorders have a wide occurrence; and the notion of anxiety, understood in relation to stress, is extremely broad. Several hypotheses indicate the implication of stress (traumatic, psychosocial, etc.) and anxiety in general vulnerability to mental disorders, through complex repercussions on a subject’s cerebral and affective development, and on his capacity to adjust to the surrounding environment (Elzinga et al., 2008; Pine, 2009).

In subjects suffering from 22q11.2 microdeletion, the frequent comorbidity of psychiatric disorders could corroborate this non-specificity. Nor is it certain that potential biomarkers discovered on the basis of this syndrome would turn out to be more specific: generally speaking, genetic risk factors highlighted in psychiatry are rather general than specific to a particular mental disorder (Kendler, 2005).

Does this mean that certain forms of anxiety disorders and anxiety are more specific than others to risk of psychosis? Clinical research carried out on patients afflicted with 22q11.2 microdeletion does not seem to offer a definitive answer. Several papers point to the predominant role of specific phobias or social anxiety as predictive factors for schizophrenia (Gothelf et al., 2013; Schonherz and Davidov, 2014), but many different forms of anxious disorders categorized by the DSM can be observed in these children to varying degrees: specific phobias, social anxiety disorder, generalized anxiety disorder, separation anxiety disorder, as well as obsessive-compulsive disorders (Feinstein et al., 2002; Jolin et al., 2009).

While the majority of these studies rely on DSM typology, a psychoanalysis-oriented differential psychopathology could provide a relevant contribution by describing different forms of anxiety, helping to narrow down the specific role of anxiety as a risk factor for psychosis.

While the notion of risk factor is based on establishing correlations, a psychoanalytical approach emphasizes the psychodynamic role of anxiety in the transition to psychotic symptoms. This point is crucial in the early prevention targeted by vulnerability studies: therapeutic treatment requires to explore the transition between anxious symptoms and psychotic symptoms.

22q11.2 microdeletion syndrome appears to highlight the interest of such a psychodynamic view, because of its complex clinical interactions between organic and mental disorders. Anxiety in these children could be the psychological result from somatic, cognitive and relational disturbances experienced during their development (Beaton and Simon, 2011). Several authors underline a vicious cycle: anxiety amplifies difficulties in communication, social avoidance, and low self-esteem, and hamper the child’s mechanisms of coping with the difficulties generated by his handicap (Angkustsiri et al., 2012).

A psychoanalytical approach focused on symptom dynamics and functions could be fruitfully applied to the interaction between anxiety and relational-cognitive difficulties. It can thus bring about hypotheses on the relations between anxiety and onset of psychosis.

We focus on these points, by drawing on the specifically psychoanalytical approach to anxiety.

## Psychoanalytical Psychopathology of Anxiety in 22q11.2 Microdeletion Syndrome

Clinical research on anxiety as a risk factor for psychosis is mostly based on evaluating anxious symptoms and disorders. However, psychoanalytically speaking, anxiety does not stem solely from a symptom or a mental disorder: rather, it initiates a process shaping various different symptoms – even when not explicitly manifest in them – by triggering Ego defenses (Freud, 1926/1959). In this sense, the psychical function of a symptom always refers back to early Ego defenses against anxiety – by displacement, inhibition, or even splitting.

Therefore, this perspective leads to question potential vulnerability to psychosis from the perspective of these different types of anxiety, which do not necessarily assume the aspect of a symptom or an anxious disorder: castration anxiety, object-loss anxiety, anxiety of destruction or fragmentation, and so on. While these different modalities of anxiety can coexist in a subject, mental functioning is most often organized around one dominant modality, a decisive element for differential clinical practice in psychoanalysis. Each modality corresponds to a type of object relation, psychical conflict, and defense mechanism, preferentially organizing a subject’s psyche and his link to the environment (Bergeret, 2013). Thus, one symptom can indicate different mental organizations and psychopathological diagnostics, depending on the underlying type of anxiety: social anxiety can express an inhibition resulting from castration anxiety, an avoidance engendered by object loss anxiety, or even an autistic withdrawal in the face of annihilation anxiety.

### The Importance of Early Anxieties in Children With 22q11.2 Microdeletion Syndrome

While anxious symptoms are diverse in 22q11.2 microdeletion syndrome, psychoanalysis invites to explore the predominance of certain types of anxiety subjacent to their symptoms in the development of these children. Notably, certain forms of anxiety, which always play a role in psychical development, could prove more difficult to elaborate and overcome because of the neurodevelopmental difficulties specific to these children.

These difficulties arise early in development, during the phase of primary narcissism, when the psyche is constituted through primary identification from its intertwining with the corporeal dimension – which Freud initially theorized in terms of the unification of autoerotic drives (Freud, 1914/1957, 1923/1961). Children afflicted with 22q11.2 microdeletion often face repeated surgery, periods of hospitalization which entail experiences of early separation from their families and close ones, anomalies in deglutition that complicate eating and everyday life, and difficulties in vocal articulation and communication. How does the subjectivity of these children, during its constitution, cope with psycho-corporeal anxieties typical of early developmental phases? One thinks of autistic dismantling anxiety (Meltzer, 1975/2008), destruction and infinite falling (Winnicott, 2016), becoming indistinguishable and then abandoned -, and anaclitic depression (Spitz, 1945). As Post-Freudian psychoanalysis has shown, the meaning given to childhood anxieties by the surrounding environment largely conditions (and supports) primary identification. Several theories have sought to specify this support (Winnicott, 2016; cf. e.g. Putois, 2015). For Lacan (1949/2006; 1962–1963), the name given to the infant by the Other allows for the constitution of a unified body image: this primary identification through speech limits the experience of bodily fragmentation due to partial drives, a source of archaic anxieties. For Winnicott – familiar with Lacan’s work – the reflexivity of the mothering environment, enabling it to distinguish their own lived experiences from those of the distressed infant, enables the latter’s introjection of a containing environment.

The situations faced by families these children frequently give rise to traumas correlative with separation, diagnostic diagnostic odyssey, and more generally attacks on family narcissism (Mannoni, 1973, 1987; Potier et al., 2016). This makes primary identification more difficult, and subsequently the containment of the child’s anxieties. This can in turn affect his interactions with his or her peers, and thereby reinforce narcissistic fragility (Potier et al., 2016): these difficulties in identification and the fragility of his self-confidence weakens his defenses against the anxiety emerging in those situations.

### Psychodynamic Hypotheses on Transition From Anxiety to Psychosis

The differential clinical practice of psychoanalysis provides a contribution to discussions on a specific vulnerability to psychosis, by highlighting the psychical processes involved. The psychoanalytic hypothesis that certain types of anxiety would more likely be linked to psychotic functioning lead to examine the singular meaning and role of anxiety in the psychical economy of a single subject. Dynamic links between anxiety and the onset of psychotic symptoms provide elements allowing to envision the risk of psychosis in 22q11.2 microdeletion syndrome, and its clinical prevention.

Firstly, psychotic symptoms may possess a function of defense against the anxiety provoked by certain traumatic elements that have forcefully entered the psyche and which cannot be metabolized and elaborated (Bion, 1956; Aulagnier, 2003). Work on clinical practice with psychoses and early stages of child development (Klein, 1946) has emphasized primitive, archaic anxieties – destruction, devouring, and fragmentation – which summon defense mechanisms similar to those encountered in adult psychoses: splitting of ego and/or object, denial, or even delusional projection. In schizophrenic patients, hallucinations, delusion, or social withdrawal can thus appear as a defensive rampart against the resurgence of early anxieties or trauma – just like the infant’s early anxieties – which cannot be integrated and subsequently fracture and divide the psyche.

We believe that Freud’s conception of anxiety is complementary to that of studies stressing the neurobiological effects of early stress or trauma on vulnerability to psychosis (Freeman and Fowler, 2009; Beaton and Simon, 2011): this may be linked to sediments of ancient traumatic events, referring to the infant’s first states of distress and powerlessness. Therefore, the focus is at the intersection between biology and the psyche - not distinct at this stage (Freud, 1926/1959; Winnicott, 1960).

This complementarity seems to fit well with 22q11.2 microdeletion syndrome: several authors maintain that the medical, cognitive and relational difficulties of these children could be acting as psychological traumas (Freeman and Fowler, 2009; Angkustsiri et al., 2012, 2014; Schonherz and Davidov, 2014). In non-pathological development, the early forms of bodily distress and anxiety can be gradually assimilated when the surrounding environment is sufficiently containing during the phase of primary identification: functioning as a protective shield against intrusions from the outside world and from the inner thrust of the drive, it allows the child to learn and tolerate archaic anxieties, and subsequently punctual frustrations and separation (Winnicott, 1953; Lacan, 1949/2006; Vanheule, 2011). By weakening this containing function, neurodevelopmental handicap weakens its shielding effect: this can lead children faced with intense anxieties to turn to defense mechanisms such as denial, possibly including delusional projection or autistic withdrawal. It should be kept in mind that some of these children experience psychotic symptoms very early (Debbané et al., 2006).

Secondly, a psychodynamic understanding of anxiety would require to examine the subjective functions of anxious disorders, especially during clinical follow-up. They can appear as attempts to localize anxiety – notably, in a phobic object – and channel it (e.g., obsessions and compulsions). In this sense, anxious symptoms can constitute an attempt at figuring and externalizing archaic anxieties or mnemonic traces of such early distress: these symptoms could, therefore, be a protective attempt of the psyche, striving to avoid turning to psychotic defenses. These psychodynamic hypotheses constitute important theoretical and clinical elements for the construction of the therapeutic alliance (transference) and treatment, aiming at opening up the path to other defensive resources that less harmful to socialization and self-esteem than anxious disorders.

## Conclusion and Perspectives

Our perspective is that of complementarity between the psychoanalytical, neurodevelopmental, and psychiatric paradigms (Bazan, 2011; Milrod et al., 2014; cf. also, on necessary complementarity, Armando et al., 2017, 2018). Such complementarity is necessary to research on vulnerability to psychosis, and conditions the reopening of the dialogue between psychoanalysis and psychiatry – which, as Rudden et al. (2003) have underlined, has almost ceased due to the gap between their respective paradigms. Reestablishing this dialogue indeed requires “psychodynamic models of psychological diatheses toward certain illnesses, or of dynamic constellations commonly set in motion by these illness processes themselves” (op. cit., 997).

The different facets of 22q11.2 microdeletion syndrome highlight the clinical requirements for this interaction between approaches. Psychoanalytic contributions such as the one we tried to sketch out would invite to explore several different perspectives regarding both therapeutic treatment of these children and adolescents, and research into early detection of psychosis.

The hypothesis of vulnerability to psychosis correlated with anxious disorders in these subjects shows the importance of longitudinal clinical follow-up, and of psychotherapeutic treatment of anxiety from childhood onward (for a psychoanalytic take on vulnerability to psychosis, see Masi, 2018’s important 2018). The decisive place that psychoanalytical hypotheses – especially Post-Freudian – ascribe to early interactions and the constitution of narcissism leads to a specific approach to the difficulties associated with this neurodevelopmental syndrome. It also examines the impact of physiological dysfunctions and medical interventions faced by these children, on the constitution of bodily unity, self-esteem, and processes of identification. Recent research (Kates et al., 2019) has shown the predictive impact of family conflict on anxiety disorders: clinical research on early interactions is therefore an important preventive direction.

The psychodynamic perspective can also contribute to early detection of anxiety and, consequently, of potential vulnerability to psychosis. In this respect, we should explore the relevance of psychoanalytical methods and tools with respect to differential diagnosis and clinical prognosis (Louët et al., 2010). Projective techniques notably the Rorschach test, can enable a fine identification of different types of anxiety, and open up hypotheses on risk factors for psychosis (Wood et al., 2000; Kimhy et al., 2007). Similarly, in psychoanalytical diagnosis, the crucial role of transfero-transferential dynamics – especially as an echo of archaic anxieties – invites to develop specifically targeted assessment tools, along with treatment guidelines. This could be expanded in a promising avenue targeting observable signs: in such children, would the types of anxiety more typical of psychotic mental functioning (from a psychoanalytic, countertransferential standpoint) match different attachment profiles – say, disorganized vs. insecure attachment? We have no *a priori* position on this point: a blind double assessment (type of anxiety vs. attachment profile) would be required to that effect. But in any event, such a research could provide clinicians with qualitative, fine-grained profiles potentially helping for early detection and prevention.

Research on the intersection between 22q11.2 microdeletion syndrome and on vulnerability to psychosis questions the psychoanalytical approach and leads to enrich its hypotheses: on the one hand, on the psychical and relational impact of somatic and cognitive disturbances, and on the other hand, on the tools for fine-tuning a differential clinical practice at the intersection between neurological and psychiatric registers.

## Author Contributions

RP decided to focus on anxiety our approach to 22q11.2 microdeletion syndrome, co-designed the structure of the argument, supervised the bibliographical research, drew on his epistemological and clinical work on comparative diagnosis, and wrote the final version of the manuscript. ST wrote a partial first draft under OP’s supervision, drew on her epistemological and clinical research on models of psychotic vulnerability, and co-designed the structure of the argument. OP drew on his work on therapeutic settings for patients afflicted with multifactorial genetic diseases (including 22q11.2 microdeletion syndrome), and supervised the structure of the argument. All authors have agreed to the final version of the manuscript.

## Conflict of Interest

The authors declare that the research was conducted in the absence of any commercial or financial relationships that could be construed as a potential conflict of interest.
